# Panic Food Purchasing amid COVID-19 Pandemic: Does the Impact of Perceived Severity, Anxiety and Self-Isolation Really Matter?

**DOI:** 10.3390/ijerph192215277

**Published:** 2022-11-18

**Authors:** Abu Elnasr E. Sobaih, Fatheya Moustafa

**Affiliations:** 1 Management Department, College of Business Administration, King Faisal University, Al-Ahsaa 31982, Saudi Arabia; 2 Faculty of Tourism and Hotel Management, Helwan University, Cairo 12612, Egypt

**Keywords:** anxiety, consumer buying behaviour, COVID-19, panic food purchasing, perceived severity, post pandemic behaviour, self-isolation

## Abstract

This research examines the influences of perceived severity, anxiety, and self-isolation intention, amid the coronavirus disease of 2019 (COVID-19), on panic food purchasing. The research adopted a quantitative approach using a pre-examined instrument, which was self-administered by the research team (with support from a data collection-specialised company) to consumers who were urgently shopping for food in the Kingdom of Saudi Arabia (KSA). The results of structural equation modelling (SEM) using analysis of a moment structures (AMOS) software showed a significant positive impact of perceived severity on consumers’ anxiety and self-isolation intention amid the COVID-19 pandemic. Self-isolation intention was found to have a significant positive impact on the anxiety of consumers amid the pandemic. Additionally, perceived severity, anxiety, and self-isolation have a significant positive impact on panic food purchasing. Both anxiety and self-isolation were found to have partial mediating effects in the link between perceived severity and panic purchasing intention. The results of the current research contribute to a better understanding of factors that influence panic purchasing behaviour, especially amid a pandemic. This will help policymakers to deal with this behaviour when such issues arise in the future. Other implications for scholars and policy makers are discussed.

## 1. Introduction

The coronavirus disease of 2019 (COVID-19) has had an unparalleled impact on the world. The COVID-19 pandemic has also had a significant impact on the international economy, as many organizations were either totally locked down or unable to function properly due to numerous constraints by policymakers [1], responding to the guidelines of the World Health Organisation (WHO). In the same context, the patterns of consumers’ spending have been affected by the pandemic supplemented with a decline in income flow [2]. Moreover, the pandemic has had significant psychological impacts and caused anxiety globally [1,2]. Due to this epidemic, people have isolated themselves and recorded unusual purchasing behaviour [3]. Additionally, recent research studies (e.g., [4,5,6]) show that the perceived severity of a pandemic by consumers has led to stockpiling behaviour. Consumers were found to limit their store visits and purchase more products than they needed at the time of purchase [4]. To explain this, some interesting examples included people buying fresh meat and saving it in their freezers due to the fear that it might be inaccessible in the future. Nonetheless, this unusual purchasing behaviour is an unhealthy action since it will have an impact on other people. This stockpiling behaviour by people amid the pandemic is perceived as a form of panic buying, which has a negative impact on the economy by disturbing the market’s supply, leading to stock-outs and raising prices [7,8]. This also has a negative effect on businesses’ profit margins, especially in the long term [9]. Therefore, it is crucial to understand this panic purchasing behaviour and understand the determinants of this unusual behaviour [10].

Earlier studies on panic purchasing have addressed this behaviour through various lenses, including “policy and legal actions” [11], “retailers and suppliers” [12,13], “panic purchase of vaccines” [14], “natural disasters” [15], “social media influences” [16], and “urgency of impulsive purchase” [17]. Previous research has shown that different psychological variables such as uncertainty, perceived severity and perceived scarcity [5], cyberchondria, self-isolation, and purchase self-efficacy [6], stress [18], anxiety [5,19], and even pleasure [20] contribute to unusual and/or panic purchasing. Despite the fact that before and during the pandemic there were growing studies [5,6,7,8,21] that addressed consumers’ unusual and panic-buying behaviour, there is still a need for more understanding of unusual buying amid a pandemic. Understanding these determinants of panic food purchase will enable policymakers to better deal with such factors in any similar future situations. It will also help scholars better understand the antecedents of this panic food-buying behaviour, which will ultimately contribute to the control of such behaviour.

The current research aims to examine the influences of consumer psychological states (i.e., perceived severity, anxiety) and self-isolation intention on their panic food purchasing intention amid the COVID-19 pandemic in an under-studied country such as the Kingdom of Saudi Arabia (KSA). The government of the KSA, through the National Development Fund, determined in 2021 to commit 120 billion Saudi Riyal (approximately USD 33 billion) as a quick response to save the lives of its citizens and residents and support the sectors affected by the COVID-19 pandemic [22]. The government did make all endeavours to take care of its people’s health and make them feel psychologically safe. However, to what extent did these endeavours affect the psychological state of consumers amid COVID-19? How has the severity of the COVID-19 pandemic influenced panic purchasing behaviour? To what extent do anxiety and self-isolation affect panic purchasing? The current study examines the interrelationships among the above-mentioned factors that influenced consumer panic purchasing of food and drink amid the pandemic. Hence, policymakers can take the lessons from this to handle similar situations in the future. The findings of this study have some implications for scholars in relation to understanding panic purchasing and how it could be controlled, especially after the COVID-19 pandemic.

To achieve the above-mentioned purpose of the current study, we adopted the following structure. After highlighting the research problem and purpose in the introduction, we discussed the theoretical foundation of our study and then reviewed previous studies and the related literature to build the research hypotheses. We then moved to present the research methodology adopted in our research, i.e., the data collection method, research measure, research sample, and data analysis approach. We then presented the findings of the study and discussed these findings. We also highlighted the implications of the study for policymakers and scholars. We ended this article by presenting the conclusion and the limitations of the study.

## 2. Literature Review

### 2.1. Panic Purchasing amid COVID-19

The Oxford Dictionary [23] defines panic purchasing as “*the action of buying large quantities of a particular product or commodity due to sudden fears of a forthcoming shortage or price increase*”. Panic purchasing is a natural response by people to crises, especially if they perceive this crisis as severe and threatening [4,5]. Despite the fact that panic purchasing is a widespread phenomenon, it has gained little consideration from academics [4,21]. While some researchers (e.g., [24,25]) assumed that people should be blamed for buying more than their actual needs, other researchers (e.g., [26,27]) argued that people rarely engaged in this behaviour unless they became anxious due to crises. This is because when crises happen, they affect some people for a certain period of time [28].

Although research (e.g., [25,29]) has suggested that panic purchasing disrupts the supply of specific products, business experts and academics have noted that panic buying is not directly caused by a supply shortage, albeit the result of the anxiety and dread of some consumers. This anxiety is often due to the perceived severity of crises at certain times, and the anticipation that quantity of food could be limited. Additionally, others can affect people’s behaviour within the same group or network, known as subjective norms in the theory of planned behaviour [30]. According to prior studies (e.g., [31,32]), disturbances in the supply of products are the main driver for panic buying during natural disasters, pandemics, and prolonged strikes. Therefore, consumers’ fear of time and resources can push them to engage in unusual and/or panic purchasing.

### 2.2. Theoretical Foundation

A review of the research on panic buying behaviour (e.g., [5,6]) showed that they adopted different theories to understand this behaviour and its determinants. Behavioural inhibition system (BIS) theory, [33], and expectancy theory [34] were adopted by earlier scholars [5,6] to analyse this behaviour among consumers amid COVID-19. The BIS theory has been adopted mainly in neuroscience, while the expectancy theory has been mainly used in human resources in order to understand individuals’ motivations. According to the BIS theory, people experience anxiety because of an aversive stimulus, which prevents them from acting naturally [35]. Additionally, if there are any unpleasant stimuli, people become anxious, which makes them re-arrange their situation to become less anxious as much as they can [36]. On the other side, the expectancy theory includes three key elements: expectancy, instrumentality, and valence. The theory explains that people are motivated to take the required precautions to avoid the fear stimulus by their expectation of danger and their sensitivity to this dangerous object [37]. It is also argued that the fear of people may vary depending on their anticipation of bad or unpleasant results that are associated with anxiety [37]. As a result, it is crucial to think about how response expectancy can help someone avoid anxiety [38]. The BIS theory shows people’s reactions differ in various situations, whereas the expectancy theory explores how the sensitivity of anticipated fear objects affects people’s anxiety and psychological state. Despite the fact that these two theories are typically used in health settings, they can be used to understand people’s panic purchases [8,39]. Drawn on the theoretical baseline, people with BIS emotion systems may react anxiously to stimuli such as the anticipation of limited food, which could push people to panic purchasing. Additionally, it could be argued that the fear of limited supply and the severity of COVID-19 could encourage people to engage in unusual buying.

In this research, we draw on protection motivation (PMT) theory [40]. The theory was widely used to understand an individual or consumer responses to fear appeal. The theory argues that individuals or consumers protect themselves by two major factors: threat appraisal and coping appraisal. The first factor, “threat appraisal”, refers to the severity of the situation, whereas the second factor, “coping appraisal”, refers to how consumers respond to this situation. In other words, consumers first assess the severity of the situation and then adopt a coping mechanism to prevent or reduce the threat, which is referred to as “response efficacy” in PMT [40]. The theory is acknowledged for use for analysing people’s responses and behaviour amid the pandemic [5,6]. In this study, we adopted PMT to understand to what extend the perceived severity of COVID-19 affected Saudis’ anxiety and their coping with self-isolation, which ultimately affected their panic purchasing intentions. Hence, we draw on the PMT to understand how consumers in the KSA perceived the pandemic, their responses to it, and the impact of this on their panic food purchase amid the pandemic and in similar situations in future.

## 3. The Research Model and Hypotheses

### 3.1. The Relationship between Perceived Severity, Anxiety, and Panic Purchasing

Perceived severity is people’s feeling of a risk of unfavourable outcomes when they engage in or avoid a certain action [8]. Anxiety levels can be raised by personal assessments of severity [40]. This is due to the fact that when an individual faces risk or uncertainty, he or she automatically thinks to what extent this is serious [41,42]. As PMT implies, people often take precautionary action if they perceive the severity of a threat, to avoid unpleasant feelings [43]. Therefore, people could make more purchases in order to get rid of negative emotions such as a decreased sense of security, and stress [44,45].

People will seek to escape from a dreaded scenario if they anticipate a bad outcome and believe the situation may be severe, according to the expectancy theory [37]. Indeed, people become worried and engage in panic purchases due to the anxiety of standing in line for a long time or the possibility of regretting not purchasing a product [46]. There is no doubt that the COVID-19 pandemic has posed a threat to human life. This has made them feel more anxious and engage in unusual spending [7]. When a pandemic occurs, people perceive a risk of getting the disease, which may lead them to participate in the panic buying of safety products in an effort to shield themselves from potential danger and take precautions [8]. There were various videos and photos on different media sites, which confirmed a change in consumer behaviour amid the COVID-19 pandemic, such as panic buying among grocery consumers of food and toilet paper [5]. Such actions affected supply and made many shops run out of items [47]. The PMT reinforces such arguments that the perceived severity of COVID-19 made many people feel anxious and they were forced to cope with self-isolation, which ultimately affected their panic purchasing intentions [4]. Hence, we could argue that:

**Hypothesis 1** **(H1).** *The perceived severity of the COVID-19 pandemic has a positive relationship with consumer anxiety.*

**Hypothesis 2** **(H2).** *The perceived severity of the COVID-19 pandemic has a positive relationship with consumer panic purchasing intention.*

**Hypothesis 3** **(H3).** *Anxiety has a positive relationship with consumer panic purchasing intention.*

### 3.2. The Relationship between Self-Isolation and Anxiety

The COVID-19 pandemic affected the whole world and had an unparalleled impact on many countries. People’s behaviours changed because of the possibility of a threat, which had a significant impact on communication becoming contactless. Due to uncertainty, people became more worried [48,49], stressed, and confused [50]. All people, of different genders and ages, experienced negative emotions, anxiety, and loneliness as a result of isolation and restrictions brought on by quarantine [48,51]. Policymakers in most countries, with WHO support, advised people to stay at home and avoid contact with others. Scholars investigated how the epidemic impacted mental health [52,53] and encouraged people to find creative ways to cope with isolation [54]. Indeed, losing one’s routine and having fewer social interactions can lead to boredom and a feeling of isolation. These emotions distress people and increase their risk of developing mental disorders such as anxiety [55,56]. Based on these arguments, we could hypothesize that:

**Hypothesis 4** **(H4).** *Self-isolation has a positive relationship with consumer anxiety.*

### 3.3. The Relationship between Perceived Severity and Self-Isolation Intention

Because the virus spreads through direct contact with infected humans, isolation has become a critical preventative action [57]. Wilder-Smith and Freedman [58] identified four different types of actions to control COVID-19 spread: “isolation, quarantine, social distancing, and community containment”. Isolation is the term for being cut off from social contact on a personal level. A quarantine is a period during which a person or group refrains from moving around or making social contact. Social distancing refers to a more drastic action, such as closing schools to reduce human direct interaction [58]. Community containment, which is out of an individual’s control, is the total lockdown of a designated area. Community containment is often authorized by policymakers.

The term “self-isolation” refers to the intended decrease in direct interaction with other humans and avoiding crowded places such as shops and public transportation. For those who are socially engaged, engaging in this action is painful. However, it is natural that people’s perceived severity has been suggested to affect their intention to self-isolate [59]. According to PMT, a more accurate threat assessment by an individual results in immediate preventive action [40]. Additionally, it was found that perceived severity leads people to adopt proactive actions such as self-isolation [60,61]. Based on these arguments, we could hypothesize that:

**Hypothesis 5** **(H5).** *The perceived severity of the COVID-19 pandemic has a positive relationship with self-isolation intention.*

### 3.4. The Relationship between Self-Isolation and Panic Purchasing Intention

Amid the COVID-19 epidemic, people were directly or indirectly required to spend some time in quarantine. This was because policymakers were imposing several restrictions in an effort to contain the pandemic [62]. Furthermore, people had reason to prepare for such action because organizations such as the WHO had advised countries and their individuals to isolate themselves [57]. This research expects that several panic purchases were undertaken in anticipation of self-isolation. Fears of global supply chain disruptions [63] may have contributed to this further increase. It was found that people engage in unusual buying due to their anticipation of disrupted supplies or involuntary stays at home for an unknown time, which leads to the purchase of products in large quantities that are of no use to them. We could argue that if individuals want to self-isolate themselves for certain reasons outside of their control, this increases their intention to engage in panic buying. Based upon these arguments, we hypothesize that:

**Hypothesis 6** **(H6).** *Self-isolation intention has a positive relationship with consumer panic purchasing intention.*

### 3.5. The Mediating Effects of Self-Isolation and Anxiety in the Link between Perceived Severity and Panic Purchasing Intention

Previous research [40] has confirmed that perceived severity is a predicator of anxiety, whereas the anxiety levels can be raised by personal assessments of the severity of the situation. It was also well documented that people often take precautionary action if they perceive the severity of a threat to avoid unpleasant feelings [43], which is the case with COVID-19. Indeed, COVID-19 has posed a threat to human life. This has made them feel more anxious and engage in unusual spending [7]. This has also had several impacts on people’s mental health [52,53] and made them find creative ways to cope with isolation [54]. One of these coping behaviours was self-isolation as identified by Wilder-Smith and Freedman [58], which led people to have limited engagement in direct interaction with other humans and to avoid crowded spaces.

Recent research by Omar et al. [5] confirmed a full mediating effect of anxiety in the relationship between perceived severity and panic purchasing behaviour amid the COVID-19 pandemic. Despite the confirmation of the direct effects of perceived severity in self-isolation and panic purchasing amid the COVID-19 pandemic [6], the indirect effect is not yet confirmed. The current research takes this novel attempt to examine this indirect relationship. Drawn on the PMT [40], this research argues that perceived severity of COVID-19 food consumers for anxiety and their coping with self-isolation, which ultimatly affected their panic purchasing intentions. Therefore, people engage in unusual buying due to their anticipation of disrupted supplies or being forced to involuntarily stay at home for an unknown time, which leads to the purchase of products in large quantities that are of no use to them

**Hypothesis 7** **(H7).** *Self-isolation intention has a mediating effect in the relationship between perceived severity and anxiety.*

**Hypothesis 8** **(H8).** *Self-isolation intention has a mediating effect in the relationship between perceived severity and consumer panic purchasing intention.*

**Hypothesis 9** **(H9).** *Anxiety has a mediating effect in the relationship between perceived severity and consumer panic purchasing intention.*

**Hypothesis 10** **(H10).** *Self-isolation intention and anxiety have a serial mediating effect in the relationship between perceived severity and consumer panic purchasing intention.*

**Figure 1:**
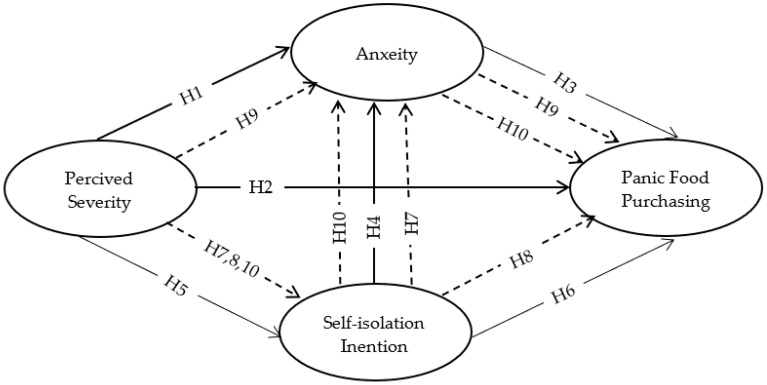
The research conceptual model; “dotted lines refer to indirect relationships”.

The research model connecting all the hypotheses is displayed in Figure 1. The model has four constructs and nine research hypotheses. Six research hypotheses examine the direct relationships, and three examine the indirect relationships.

## 4. Methodology

### 4.1. Research Population and Sample

The research population of the current study included food consumers in the KSA. The data were collected during the first quarter of 2021. At this time, there was partial closure of most stores, except food shops; otherwise, people were self-isolated at their homes. The government allowed consumers to go out only for food during certain times or for other emergencies, while maintaining social “place” distance. We respected these guidelines of distance in our data collection from participants. Hence, our data were collected with support from a company specialized in data collection. The questionnaire forms were given to participants at different food stores in the main cities of the KSA who agreed to participate in the study. We discussed the purpose of the study with participants and received their consent to participate in the study. We adopted the research sample framework of Krejcie and Morgan [64], which suggested that a population of one billion or above should have 384 or above participants in the sample. We distributed 600 forms to a random sample of visitors in the shopping malls. We were able to collect 430 valid forms for data analysis with a response rate of 71.7%. This good response rate was due to the support of the specialized data collection company and the presence of the research team members.

### 4.2. Research Measurement

We adopted pre-tested measures to examine the effects of perceived severity, anxiety, and self-isolation on panic food purchasing. We examined perceived severity through a 3-item scale from Omar et al. [5]. These 3 items were developed by other research [65,66]. An example of these items is the “COVID-19 pandemic is life-threatening”. We examined anxiety through 5 items from [5]. These items were drawn from other research studies [67,68,69]. An example of these items is “When shopping for food, I get in a state of tension or turmoil as I think over my recent concerns and interests”. We examined self-isolation through 4 items from [6], which was originally developed by [61]. An example of these items is “deliberately cancel or postpone a social event, such as meeting with friends, eating out, or going to a sporting event”. Finally, we examined the intention of food purchase through 3 items from [5]. These three items were drawn from previous research studies [70,71,72]. An example of these items is “While shopping for food, I have bought more products than what I intended to buy”. Please see Table 1 for full items of the research measure. After the questionnaire development, we conducted a pilot test with 15 professors in the college of business administration to ensure its face and content validity. We did not make any changes to the questionnaire based on the peer comments.

### 4.3. Data Analysis Technique

We adopted the statistical package for social science (SPSS) (v.25) with analysis of a moment structures (AMOS) for data analysis. We performed principal component analysis (PCA) to simplify high-dimensional data. We were able to confirm the unidimensionality of our variables, which were 55.216%, 51.670%, 50.331%, and 63.111% for perceived severity, anxiety, self-isolation intention, and panic food purchasing intention, respectively, of the total variance explained. We conducted Kaiser–Meyer–Olkin “KMO” to test the strength of the partial correlation between variables. The results showed that the values of KMO were more than 0.5 and close to 1, which meant that they were ideal [73]. The values were 815, 0.765, 0.881, and 0.805 for perceived severity, anxiety, self-isolation intention, and panic food purchasing intention 0, respectively. We were able to reject the null hypothesis, since the *p* value was 0.000. We adopted Cronbach’s Alpha to check the reliability of our measure. The findings of the Alpha were 0.991, 0.990, 0.916, and 0.898, respectively, confirming that the Alpha values were excellent [74].

## 5. Results

### 5.1. The Profile of Respondents

We distributed our questionnaire forms to a random sample of consumers at the different shopping malls in the main cities of the KSA. The profile of participants included 310 male participants (72.10%) and only 120 female participants (27.90%). The low participation of females in data collection, especially in public sectors, is difficult in the KSA due to gender segregation, which limits access to women. With regard to the age of participants, there was participation for different ranges of age. There was no participation in this research from people under 18 years old. Participants between 30 and 50 years old were the majority (45%), followed by those over 50 years old (33%) and then by those less than 30 years old (22%). All participants in this research had at least a secondary school diploma or equivalent. The vast majority had a university degree (70%). This was followed by those who held postgraduate degrees (21%) and finally those who had a secondary school diploma or equivalent (9%).

### 5.2. Descriptive and Factorial Results

We first analysed our data, i.e., mean and standard division. This was to check how concentrated the data were around the mean. We also adopted skewness, “the coefficient of symmetry”, and kurtosis, “the coefficient of flattening”, to ensure the normal distribution of our data [75,76]. The results in Table 1 of minimum, maximum, mean, standard deviation, skewness, and kurtosis confirm that our data have a normal distribution. We then conducted confirmatory factor analysis (CFA), since we adopted a pre-tested instrument to verify the items or factors fit together to measure our variables and then were able to examine the relationships. We checked the GoF, “Goodness of Fit”, in order to ensure that our collected data fit the model.

The findings of our CFA showed some evidence that ensured the convergent validity of our measures. First, the standardized loading was between 0.702 and 0.977 with a significant *p* value of “0.001”, which was above the value of 0.50 as recommended by [73]. We checked CR, “composite reliability”, to ensure the items adopted for measurement were related to the latent variable and AVE, “average variance extracted”, to ensure the variance in the construct. The results in Table 2 show that all CR values were above 0.7 and AVE values were above 0.6, which confirms the convergent validity [74] (See Table 2). The CR was 0.956 for perceived severity, 0.979 for anxiety, 0.980 for self-isolation intention, and 0.905 for panic food purchasing. The AVE was 0.879 for perceived severity, 0.903 for anxiety, 0.926 for self-isolation intention, and 0.764 for panic food purchasing (Table 2). The findings of the CFA also ensured the discriminant validity of our measures through MSV “maximum shared variance” values, which have to be lower than the squared root scores of AVE values. This was the case in our research, as shown in Table 2 in the bold scores.

### 5.3. Structural Equation Modelling Results

**Figure 2:**
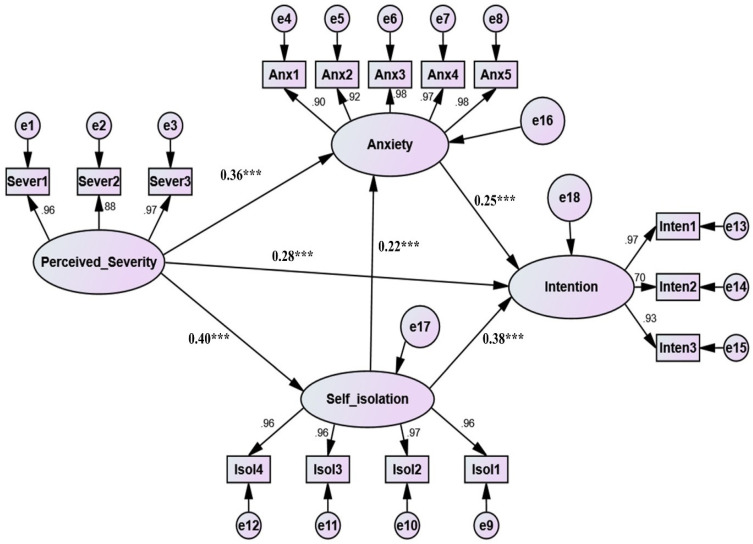
The structural model (*** *p* < 0.001.).

We adopted a confirmatory approach in our study by developing a theoretical model based on the literature review and then collecting data to examine this model via a pre-tested questionnaire form. The results of the structural model (Table 3 and Figure 2) confirm that the model has a good fit “(χ^2^ (84, N = 430) = 251.16 *p* < 0.001, normed χ^2^ = 2.99, RMSEA = 0.069, SRMR = 0.035, CFI = 0.963, TLI = 0.961, NFI = 0.967, PCFI = 0.651 and PNFI = 0.656), *** *p* < 0.001”.

Figure 2 shows the paths, which confirm/reject the research hypotheses. The results of SEM (Table 3) showed that all the direct research hypotheses were verified and showed significant relationships with *p* < 0.001 and *p* < 0.05 (Table 3, Figure 2). The results showed that perceived severity influences significantly and positively on anxiety (β = 0.36, t-value = 7.002, *p* < 0.001), self-isolation intention (β = 0.40, t-value = 8.580, *p* < 0.001), and panic purchasing (β = 0.28, t-value = 5.567, *p* < 0.001). Additionally, self-isolation intention impacts significantly and positively on anxiety (β = 0.22, t-value = 4.716, *p* < 0.001). Additionally, anxiety has a significant and positive impact on panic purchasing (β = 0.25, t-value = 5.332, *p* < 0.001). Finally, self-isolation intention impacts significantly and positively on panic purchasing (β = 0.38, t-value = 7.517, *p* < 0.01). The results also showed that the R^2^ “explanatory predictive power” of all paths (R^2^ = 0.29) accounts for about 30% of the variance in panic food purchasing.

For examining the mediating effect of both anxiety and self-isolation intention in the relationship between perceived severity and panic food purchasing intention, we adopted the approach of Zhao et al. [77,78], which has three stages. The first stage is checking the relationship between the independent and dependent variable. The second stage is checking the relationship between the independent and mediating variable. The third stage is checking the relationship between the mediating and dependent variable. If the three relationships are significant, then a partial mediation effect exists. Nonetheless, if the direct relationship between the independent and dependent variables is not confirmed and other relationships are confirmed, then full mediation exists. The results of mediation analysis, using 5000 resampling bootstrapping, support all mediation hypotheses. In this research, we found a partial mediation effect of self-isolation in the relationship between perceived severity and anxiety (see Table 4). There is also a partial mediation effect in the relationship between perceived severity and panic food purchasing intention (see Table 4). We found a partial mediation effect of anxiety in the relationship between perceived severity and panic food purchasing intention (see Table 4). There was a partial mediation of both anxiety and self-isolation (double mediation) in the relationship between perceived severity and panic food purchasing intention.

## 6. Discussion and Implications

We conducted the current study to examine the impact of perceived severity, anxiety, and self-isolation, which were results of the COVID-19 pandemic, on consumers’ panic food purchasing in the KSA—an under-studied country. More specifically, we examined the direct impact of the perceived severity of COVID-19 on consumers’ panic food purchasing and its indirect impact through anxiety, and self-isolation. We undertook factorial analyses and structural modelling using AMOS to examine our developed theoretical model and research hypotheses. The results of the SEM showed that the perceived severity of the COVID-19 pandemic on consumers had significant positive impacts on their anxiety and self-isolation intention. The long-lasting COVID-19 pandemic, with thousands of infections and people passing away on a daily basis, influenced people’s mental health disorders such as anxiety [52,53]. The uncertainty made consumers clearly perceive the severity of the pandemic, become anxious, and protect themselves by self-isolation. These findings also support the expectancy theory that people often take precautionary action if they perceive the severity of a threat and want to avoid unpleasant feelings [43]. This precautionary action includes the intention of self-isolation to protect themselves and avoid other negative consequences. People motivate themselves to cope with the threat from COVID-19 through self-isolation, which supports the PMT framework [40]. The results confirmed that self-isolation intention has a significant positive impact on anxiety. We found that when people self-isolate, they become more anxious.

The results also showed that perceived severity, anxiety, and self-isolation intention have direct and significant positive impacts on panic food purchasing among consumers in the KSA. These findings support the PMT that people reacted anxiously when they perceived the severity of the pandemic and anticipated the limitation of food, which pushed people to panic purchasing. Additionally, the fear of limited supply and the severity of COVID-19 encouraged people to engage in unusual buying. When a pandemic occurs, people perceive a risk of getting the disease, which may lead them to participate in the panic buying of safety products in an effort to shield themselves from potential danger and take precautions [8]. Unlike the results of Omar et al. [5], who found a full mediating effect of anxiety, our findings showed a partial mediation effect of anxiety in the relationship between perceived severity and panic food purchasing intention. We also found a partial mediation effect of self-isolation intention in the relationship between perceived severity and anxiety as well as panic food-purchasing intention. The results also confirmed a partial mediation of both anxiety and self-isolation intention (double mediation) in the relationship between perceived severity and panic food purchasing intention. These two variables, i.e., anxiety and self-isolation intention, were able to increase the total effect of perceived severity and panic food-purchasing intention.

Our findings have some implications for scholars. While some scholars (e.g., [24,25]) believe that consumers should be blamed for buying more than their actual needs during crises, others (e.g., [26,27]) argue that consumers do not naturally panic buy, but engage in this behaviour to react to crises. We found that panic food purchasing has become not just a required action during the months of quarantine but also a coping technique for many people in the KSA (as with other people in many countries worldwide). This behaviour is expected to continue post-pandemic if consumers perceive the severity of the situation or become anxious about any problem related to the supply chain of food. Interestingly, our research confirmed the partial mediation effects of both anxiety and self-isolation intention in the relationship between perceived severity and panic food purchasing intention, anxiety and self-isolation intention, the ability to increase the effect of perceived severity, and panic food-purchasing intention.

During lockdown periods, the local authorities in the KSA reduced the number of hours of food preparation and service locations as well as food stores. Moreover, there were other guidelines that people should avoid direct human interaction and shopping unless it was urgent. Despite the fact that their activity was for the safety of people, they caused people to become more anxious. Increased anxiety is likely to encourage consumers to purchase more items than their actual needs. Our study recommends the implementation of PMT theory to understand consumers’ buying behaviour amid crises and pandemics. Despite the support given by the government of the Kingdom to its people amid the pandemic, panic food purchasing among Saudis still exists because of perceived severity, anxiety, and self-isolation intention. The Saudis’ food consumption culture [79], which depends on buying large amounts of food more often than their needs, could have an effect on this panic buying during the pandemic, which could be an opportunity for further investigation.

The results have implications for policymakers as well. Because of their mistrust in how governments were handling the crisis, and their fear that food would run out in the near future, many consumers engaged in panic food purchasing. The KSA government did not make any endeavour to protect their people, save their lives, or ensure that all supply chain systems were running efficiently during the pandemic. However, the results of our research showed that this did not change the consumer behaviour of panic food purchasing. Therefore, media campaigns are important to ensure that people do not get anxious and have the support of the government during this crisis. These media campaigns should reduce the perceptions of COVID-19 severity, anxiety, and the negative consequences of self-isolation intention. Social media is a significant tool for influencing panic buying among consumers [16]. Hence, the government should be communicating with consumers on these social media platforms to avoid their engagement in panic purchasing behaviour.

## 7. Conclusions

This study investigated the impact of the COVID-19 pandemic on panic food purchasing intention. The study examined the relationship between perceived severity, anxiety, self-isolation intention, and panic food purchasing intention amid the COVID-19 pandemic. Our findings support the PMT framework confirming a direct and significant positive impact of perceived severity on anxiety and self-isolation intention, and the latter increases the anxiety level among consumers. The uncertainty and long-term effect of COVID-19 have maximized the level of severity among consumers, thus increasing their intention of self-isolation and their anxiety. Additionally, these three variables: perceived severity, anxiety, and self-isolation intention, were found to have a direct and significant positive impact on panic food purchasing intention amid the COVID-19 pandemic. The findings also confirmed the indirect impact of perceived severity on panic food purchasing intention through anxiety and self-isolation intention. Despite the government’s efforts to reduce the severity of the pandemic among people and decrease people’s anxiety, people still panic bought foods. This was not just because of the effects of perceived severity, anxiety, and self-isolation intention, but could also be a result of the food-consumption culture of Saudis to buy more food than their needs during normal times.

## 8. Research Limitations and Future Research Opportunities

The study was conducted on consumers in the KSA. The sample size was small (430 participants); hence, it was not representative of the whole population because we were not able to access many people due to self-isolation. Additionally, the sample was gender-biased, since about 72% of the respondents were male and only 28% were female. Furthermore, the study did not consider the moderating effect of the respondents’ demographics, e.g., gender and age, on the variables of the study, which could present a further research opportunity using Multi-group analysis for instance. Another future research opportunity could be examining the effect of food consumption culture on panic food buying, especially in countries such as the KSA, where people are culturally used to serving large portions of food for their families and guests to express generosity.

## Figures and Tables

**Table 1:** Descriptive statistics.

Abbr.	Item	Min	Max	M.	S.D.	Skewness	Kurtosis
Perceived Severity (PS) (Omar et al. [5]) (α = 0.911)							
Sever_1	“COVID-19 pandemic is a serious threat”	1	5	4.33	1.011	−1.855	3.222
Sever_2	“COVID-19 pandemic is critical”	1	5	4.38	1.030	−1.921	3.232
Sever_3	“COVID-19 pandemic is a life-threatening”	1	5	4.13	1.206	−1.555	1.481
Anxiety (A) (Omar et al. [5]) (α = 0.920)							
Anx_1	“When shopping for food, I feel that difficulties are piling up that I cannot overcome them”	1	5	4.12	1.114	−1.350	1.189
Anx_2	“When shopping for food, I worry too much over something that really doesn’t matter”	1	5	4.08	1.151	−1.309	0.980
Anx_3	“When shopping for food, I take disappointments so keenly that I can’t put them out of my mind”	1	5	3.95	1.189	−1.163	0.585
Anx_4	“When shopping for food, I get in a state of tension or turmoil as I think over my recent concerns and interest”	1	5	3.83	1.142	−0.892	0.225
Anx_5	“When shopping for food, some unimportant thoughts run through my mind and bothers me”	1	5	3.86	1.160	−0.906	0.152
Self-isolation intention (SI) (Laato et al. [6]) (α = 0.936)							
ISol_1	“Deliberately cancel or postpone a social event, such as meeting with friends, eating out, or going to a sport event”	1	5	3.59	1.186	−0.622	−0.280
ISol_2	“Reduce and/or do not use public transportation”	1	5	4.09	1.115	−1.341	1.254
ISol_3	“Avoid going to shops”	1	5	4.07	1.185	−1.296	0.855
ISol_4	“Stay at home and study/work remotely”	1	5	4.02	1.169	−1.163	0.565
Panic food-purchasing intention (PFPI) (Omar et al. [5]) (α = 0.908)							
Inten_1	“While shopping for food, I have bought more products than what I intended to buy”	1	5	3.91	1.195	−0.962	0.074
Inten_2	“Stock up food and drink”	1	5	3.71	1.361	−0.808	−0.552
Inten_3	“Unusual purchase of food”	1	5	3.70	1.301	−0.715	−0.578

Max = maximum, Min = minimum, M. = mean, S.D. = standard deviation.

**Table 2:** Convergent and discriminant validity.

Factors and Items	SL	CR	AVE	MSV	PS	A	SI	PFPI
1- Perceived Severity (α = 0.911)		0.956	0.879	0.203	**0.938**			
“COVID-19 pandemic is a serious threat”	0.957 ***							
“COVID-19 pandemic is critical”	0.881 ***							
“COVID-19 pandemic is a life-threatening”	0.972 ***							
2- Anxiety (α = 0.920)		0.979	0.903	0.203	0.450	**0.950**		
“When shopping for food, I feel that difficulties are piling up that I cannot overcome them”	0.900 ***							
“When shopping for food, I worry too much over something that really doesn’t matter”	0.921 ***							
“When shopping for food, I take disappointments so keenly that I can’t put them out of my mind”	0.977 ***							
“When shopping for food, I get in a state of tension or turmoil as I think over my recent concerns and interest”	0.974 ***							
“When shopping for food, some unimportant thoughts run through my mind and bothers me”	0.977 ***							
3- Self-isolation intention (α = 0.936)		0.980	0.926	0.157	0.396	0.367	**0.962**	
“Deliberately cancel or postpone a social event, such as meeting with friends, eating out, or going to a sports event”	0.960 ***							
“Reduce and/or do not use public transportation”	0.974 ***							
“Avoid going to shops”	0.957 ***							
“Stay at home and study/work remotely”	0.958 ***							
3- Panic food purchasing intention (α = 0.908)		0.905	0.764	0.108	0.158	0.246	0.328	**0.874**
“While shopping for food, I have bought more products than what I intended to buy”	0.968 ***							
”Stock up food and drink”	0.702 ***							
”Unusual purchase of food”	0.929 ***							

Model fit: (χ^2^ (84, N = 430) = 251.16 *p* < 0.001, normed χ^2^ = 2.99, RMSEA = 0.069, SRMR = 0.035, CFI = 0.963, TLI = 0.961, NFI = 0.967, PCFI = 0.651 and PNFI = 0.656); *** significant level less than 0.001. Please note: SL = standardized factor loading, CR > 0.7, AVE > 0.5, MSV < AVE, √AVE is bold face diagonal.

**Table 3:** Result of direct relationships.

Hypotheses	Statement	Estimate	C-R (T-Value)	R^2^	Hypotheses Results
H1- PS → A	PS has a positive relationship with A	0.36 ***	7.002		Supported
H2- PS → PFPI	PS has a positive relationship with PFPI	0.28 ***	5.567		Supported
H3- A → PFPI	A has a positive relationship with PFPI	0.25 ***	5.332		Supported
H4- SI → A	SI has a positive relationship with A	0.22 ***	4.716		Supported
H5- PS → SI	PS has a positive relationship with SI	0.40 ***	8.580		Supported
H6- SI → PFPI	SI has a positive relationship with PFPI	0.38 ***	7.517		Supported
PFPI				0.29	

Model fit: (χ^2^ (84, N = 430) = 307.104 *p* < 0.001, normed χ^2^ = 3.656, RMSEA = 0.028, SRMR = 0.0414, NFI = 0.901, CFI = 0.913, TLI = 0.915, PCFI = 0.650 and PNFI = 0.646), *** *p* < 0.001.

**Table 4:** Result of indirect relationships.

Path	Indirect (a,b)	Direct (c′)	Total (c)	Mediation
H7- PS → SI → A	0.124 **	0.360 ***	0.484 ***	Partial mediation Supported
H8- PS → SI → PFPI	0.143 **	0.280 ***	0.432 ***	Partial mediation Supported
H9- PS → A → PFPI	0.151 **	0.280 ***	0.431 ***	Partial mediation Supported
H10- PS → (SI → A) → PFPI	0.103 **	0.280 ***	0.383 **	Partial mediation Supported

** *p* < 0.01, *** *p* < 0.001.

## Data Availability

Data are available upon request from researchers who meet the eligibility criteria. Kindly contact the first author privately through the e-mail.
